# Dose reduction in dynamic synaptic vesicle glycoprotein 2A PET imaging using artificial neural networks

**DOI:** 10.1088/1361-6560/ad0535

**Published:** 2023-12-08

**Authors:** Andi Li, Bao Yang, Mika Naganawa, Kathryn Fontaine, Takuya Toyonaga, Richard E Carson, Jing Tang

**Affiliations:** 1 Department of Biomedical Engineering, University of Cincinnati, Cincinnati, OH, United States of America; 2 School of Biomedical Engineering, Southern Medical University, Guangzhou, Guangdong, People’s Republic of China; 3 Positron Emission Tomography Center, Department of Radiology and Biomedical Imaging, Yale University, New Haven, CT, United States of America

**Keywords:** dose reduction, artificial neural network, dynamic PET, SV2A imaging with ^11^C-UCB-J, spatiotemporal denoising

## Abstract

*Objective*. Reducing dose in positron emission tomography (PET) imaging increases noise in reconstructed dynamic frames, which inevitably results in higher noise and possible bias in subsequently estimated images of kinetic parameters than those estimated in the standard dose case. We report the development of a spatiotemporal denoising technique for reduced-count dynamic frames through integrating a cascade artificial neural network (ANN) with the highly constrained back-projection (HYPR) scheme to improve low-dose parametric imaging. *Approach*. We implemented and assessed the proposed method using imaging data acquired with ^11^C-UCB-J, a PET radioligand bound to synaptic vesicle glycoprotein 2A (SV2A) in the human brain. The patch-based ANN was trained with a reduced-count frame and its full-count correspondence of a subject and was used in cascade to process dynamic frames of other subjects to further take advantage of its denoising capability. The HYPR strategy was then applied to the spatial ANN processed image frames to make use of the temporal information from the entire dynamic scan. *Main results*. In all the testing subjects including healthy volunteers and Parkinson’s disease patients, the proposed method reduced more noise while introducing minimal bias in dynamic frames and the resulting parametric images, as compared with conventional denoising methods. *Significance*. Achieving 80% noise reduction with a bias of −2% in dynamic frames, which translates into 75% and 70% of noise reduction in the tracer uptake (bias, −2%) and distribution volume (bias, −5%) images, the proposed ANN+HYPR technique demonstrates the denoising capability equivalent to a 11-fold dose increase for dynamic SV2A PET imaging with ^11^C-UCB-J.

## 1. Introduction

Positron emission tomography (PET) serves as a diagnostic imaging modality to quantitatively measure physiological and biochemical processes *in vivo*. Its clinical contribution has been demonstrated in oncologic (Delbeke 1999, Bomanji *et al* 2001, Gambhir 2002), cardiologic (Plein and Sivananthan 2001, Schindler *et al* 2010) and neurologic (Slifstein and Abi-Dargham 2017, Kreisl *et al* 2020) applications. Static PET imaging provides an activity image of radiotracer distribution from reconstructing coincidence events accumulated over a certain scan duration. Dynamic PET bins the events into time frames of shorter durations to track the spatiotemporal distribution of a tracer. Parametric images, reflecting the uptake rate of a tracer from plasma to tissue or the tracer’s distribution volume, can be estimated by applying kinetic modelling to time activity curves (TACs) extracted from the dynamic frames (Carson 2005). Taking advantage of the temporal information in tracer kinetics, dynamic PET imaging adds value in supporting disease diagnosis and treatment monitoring (Takesh 2012, Dimitrakopoulou-Strauss *et al* 2021).

Synaptic vesicle glycoprotein 2A (SV2A) is a vesicle membrane protein ubiquitously and homogeneously located in synapses throughout the entire brain (Bajjalieh *et al* 1994, Mendoza-Torreblanca *et al* 2013). It serves as a molecular target for PET imaging to specifically monitor synaptic density (Masliah *et al* 1990, Kaufman *et al* 2015). ^11^C-UCB-J was developed as a PET radioligand that binds to SV2A for non-invasive visualization and quantification (Nabulsi *et al* 2016). It has been confirmed that dynamic PET imaging of SV2A is sensitive to synaptic loss in patients with brain disorders, including temporal lobe epilepsy (Finnema *et al* 2016), Alzheimer’s disease (AD) (Chen *et al* 2018, Mecca *et al* 2020, O’Dell *et al* 2021), and Parkinson’s disease (PD) (Matuskey *et al* 2020). In addition to abnormal synaptic distribution in these diseases, changes in synaptic structure and function connectivity were detected in autism spectrum disorder (ASD) model mice (Zoghbi and Bear 2012, Tang *et al* 2014, Pagani *et al* 2021). SV2A PET imaging holds potential to further our understanding of the pathophysiology of ASD and/or help categorizing ASD subtypes beyond animal models. Administering radioactive material, however, limits the application of PET imaging in children who are more sensitive to radiation than adults. Lowering injection dose in SV2A PET studies is not only desirable for adult imaging but also imperative for opening up the possibility of using PET for brain disorder research in children, especially solving the gross disparity between the high prevalence of ASD and the number of molecular imaging (PET and SPECT) studies (Zürcher *et al* 2015).

While dose reduction confers less radiation exposure, it lowers the signal-to-noise ratio in the reconstructed images especially for dynamic PET, which divides coincidence events into multiple time frames. This would result in high levels of noise in the extracted TACs, compromising the reliability of estimated parametric images with kinetic modelling performed particularly at the voxel level. Denoising techniques have been applied or customized for parametric imaging to process reconstructed time frames before kinetic analysis to address noise while maintaining the accuracy (Gallezot *et al* 2019). Gaussian smoothing in the spatial domain is a commonly used method which reduces noise in individual frames at the cost of blurring boundaries between adjacent regions with different kinetics. Advanced approaches incorporate temporal information available in dynamic imaging to implement spatiotemporal processing aiming to better preserve the resolution. Working on the similarity between voxel TACs, methods such as anisotropic diffusion (Tauber *et al* 2011) and bilateral filtering (Bian *et al* 2014) have been adopted to take temporal and spatial consistencies of the data into consideration. Denoising techniques including wavelet processing (Alpert *et al* 2006), Gaussian filtering combined with expectation-maximization deconvolution (Floberg and Holden 2013), and non-local means (NLM) (Dutta *et al* 2013) have also been tailored to process the spatiotemporal images made of dynamic time frames or the extracted spatiotemporal patches. In addition, temporal information has been integrated in denoising procedures through generating a composite image of higher SNR from all or some of the time frames with representative examples of highly constrained back-projection (HYPR) processing (Christian *et al* 2010, Floberg *et al* 2012) and composite image guided filtering (Lu *et al* 2014). Although these methods have been demonstrated effective in suppressing noise in reconstructed image frames and/or parametric images, most of them rely heavily on smoothing parameter selection to be successful.

Benefiting from the powerful learning capability, neural network-based methods have shown their superior performance in image processing and related fields including medical imaging. The advantage of machine learning based denoising techniques lies in the ability to automatically learn the optimal internal parameters in the training process. No prior assumptions need to be made on, for example, the relationship between neighbouring voxels or noise distribution models as usually required by conventional methods. Various studies aimed to recover static standard-dose PET images from low-dose reconstructions using regression model-based (Gong *et al* 2019, Liu *et al* 2020, Schaefferkoetter *et al* 2020, Spuhler *et al* 2020) or generative model-based (Wang *et al* 2018, Zhou *et al* 2020, Gong *et al* 2021) methods. Networks in these methods are trained using large image patches extracted from sub-sampled PET images and their corresponding full-dose images. A combination of anatomical information from magnetic resonance (MR) imaging or computed tomography (CT) with the training PET images also demonstrated to improve performance of the trained networks (Xiang *et al* 2017, Wang *et al* 2019, Ladefoged *et al* 2021, Schramm *et al* 2021). Although large image patches (rather than entire images) are used as the training data, these methods still require a decent number of subjects to include adequate spatial variations for training. Moreover, generalization is important in learning-based methods which measures the capability of a trained model to process the unencountered data with the same distribution as the training data (Zhang *et al* 2021). Networks with delicate structures, however, achieve great performance at the cost of limited generalization capability. A mismatch between the training data and the testing data or the lack of well-registered MR or CT images affect the performance.

As for dynamic PET imaging noise reduction with machine learning, TACs were used as one-dimensional signals to train a multi-layer feedforward artificial neural network (ANN) model for denoising voxel-wise TACs instead of the image frames of testing data (Angelis *et al* 2021). There also have been studies using both spatial and temporal information from dynamic PET images to train the networks, such as the stacked sparse autoencoder (SAE) which consists of several encoders and a decoder (Cui *et al* 2017) and the deep denoising autoencoder (DAE) (Klyuzhin *et al* 2019). These methods have been shown to outperform conventional denoising methods in terms of certain image quality metrics. However, their specification of the training data including the number of frames and the tracer kinetics, i.e. the particular TAC shape put restrictions on their applicability. They also require large training datasets to include adequate temporal information and the trained models led to erroneous outcomes when applied to data which were not included in training.

In this paper, we propose an ANN based denoising method to achieve dose reduction for ^11^C-UCB-J dynamic PET imaging while preserving the quantitative measurement accuracy. We design and train a patch-based ANN model to form a nonlinear mapping between image patches in reduced-dose dynamic frames and their full-dose correspondences. Instead of using entire images or large image patches, a vast number of image patches with relatively small size from a frame of one subject are extracted to generate the training input, which lowers the requirement for the training datasets. Due to the small size of patches used for training and processing, fully connected layers are adopted to make full usage of the information carried in each patch. Benefiting from the generalization capability of patch-based operations, the trained ANN model is expected to be applicable to denoise image frames of other subjects. Inspired by a cascade training scheme designed to remove artifacts induced by denoising (Wu *et al* 2017), we execute the cascade strategy by applying the one-time trained ANN model multiple times to maximize its denoising capability and facilitate the investigation of its denoising property. To integrate data over the time course of dynamic studies, we then apply the HYPR algorithm (Christian *et al* 2010) to the ANN processed image frames to implement spatiotemporal denoising before kinetic modeling for parametric image estimation. We describe the proposed ANN+HYPR method including the model training and testing in section 2. The experiments performed on 10 subjects including healthy controls (HCs) and PD patients to compare the proposed method with representative existing methods are presented in section 3. We show the evaluation results of processed low-dose image frames and the corresponding parametric images in section 4. Section 5 discusses the considerations in the method development and its comparison with other methods and section 6 summarizes the contribution of this study.

## 2. Methods

We first introduce the proposed ANN model to be trained for denoising dynamic activity image frames reconstructed from reduced-count scans. We elaborate the architecture, the training data generation, and training and testing of the patch-based ANN model. We then describe the HYPR method applied to the ANN processed image series which makes the method a spatiotemporal ANN+HYPR denoising technique. The conventional denoising methods we used for comparison are briefly presented at the end of this section.

### 2.1. ANN spatial denoising

**Figure 1.**
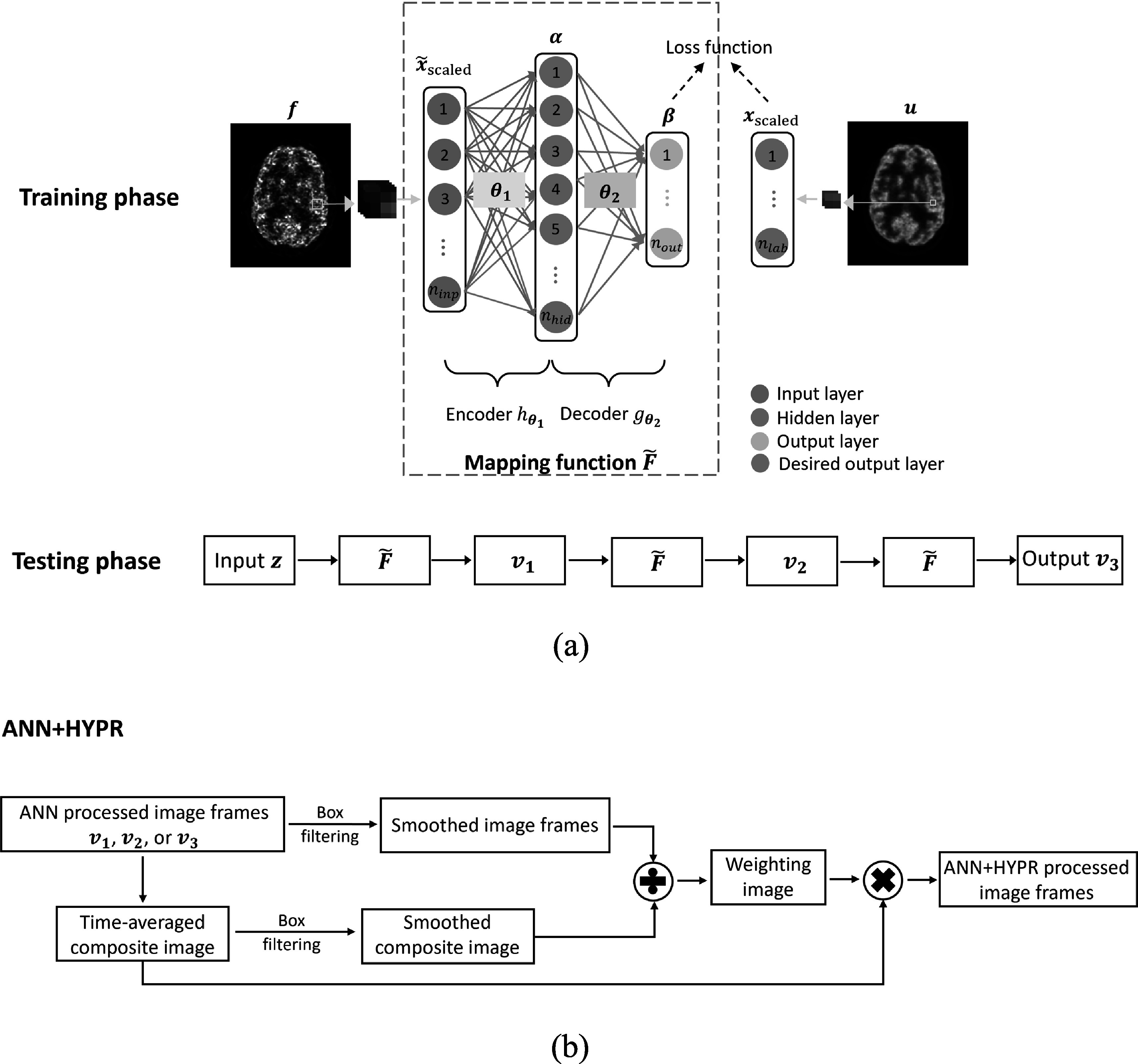
(a) Architecture of the cascade ANN model. The patches extracted from the reduced-count image ${\boldsymbol{f}}$ and corresponding full-count image ${\boldsymbol{u}}$ are normalized to form the input and the desired output vectors, respectively. ${{\boldsymbol{\theta }}}_{{\bf{1}}}$ and ${{\boldsymbol{\theta }}}_{{\bf{2}}}$ denote parameter sets. The cascade operation is implemented by feeding the latest output (i.e. ${{\boldsymbol{v}}}_{{\bf{1}}}$ or ${{\boldsymbol{v}}}_{{\bf{2}}}$) to the mapping function again. The final result is obtained after the ANN model is applied three times in the testing phase. (b) Architecture of HYPR processing of dynamic image frames after the spatial ANN processing.

The proposed ANN model includes an input layer, a hidden layer, an output layer, and a desired output layer, as illustrated in figure 1(a). This fully connected feedforward ANN is trained to form a patch-to-patch mapping from a reduced-count dynamic frame to its full-count correspondence of the training subject. The reduced-count and the corresponding full-count images are denoted as ${\boldsymbol{f}}$ and ${\boldsymbol{u}},$ respectively. Below we present the procedures to train the approximation function $\widetilde{F}$ that maps the 3D patches in ${\boldsymbol{f}}$ to the patches in ${\boldsymbol{u}}$ of a HC subject and the steps to apply $\widetilde{F}$ to denoise a new dynamic frame ${\boldsymbol{z}}$ of other HCs and PD patients.

#### 2.1.1. Training data preparation

To effectively account for the intensity differences between the training and testing data, the reduced-count image used as the training image is first normalized to [0 1] using its maximum intensity value. For a voxel location in ${\boldsymbol{f}},$ we first extract a 3D patch with 4 × 4 × 4 voxels. After subtracting its mean value from the patch, the patch voxels are stacked into a vector $\widetilde{{\boldsymbol{x}}}$ to compose a training input. We generate the label vector ${\boldsymbol{x}}$ by extracting the center of the corresponding patch with the size of 2 × 2 × 2 from the full-count image ${\boldsymbol{u}}$ and subtracting its mean value. The input patch size is chosen based on our previous brain imaging study (Yang *et al* 2018), which was demonstrated to capture fine structures and robust to noise. The smaller output size reduces the number of parameters to learn. In addition, compared with predicting a whole image patch, predicting its central section is intuitively easier. The variation of the intensity values within the central section of an image patch would be smaller than (or equal to) that of the whole patch and it is easier to mathematically model less-variant data. Reducing the output patch voxel number also serves the purpose of better maintaining the regional mean, which is further explained in section 2.1.3. We expect a smaller bias by outputting a 2 × 2 × 2 patch than a 4 × 4 × 4 patch in the training and testing phases. The patch mean value is subtracted to simplify the training task in order to learn the relationship of intensity variations between the input and label patches regardless of the absolute mean value.

Sliding the patch location one voxel at a time, we extract all possible patches covering the full region of the training input ${\boldsymbol{f}}.$ Due to the similarity among the extracted patches, we choose a representative subset by calculating the variance of each label patch and ranking the corresponding input patches accordingly. A total of 100 000 patches are selected for training with 50 000 of them having larger variance inferring larger intensity gradients or larger noise and another 50 000 randomly selected from the remaining patches that might include uniform ones. This number of training patches is decided based on the unknown parameters in the proposed model described in the next subsection to mitigate the risk of overfitting as well as underfitting that could happen in the case of using all the extracted patches. This variance-based training patch selection method has been demonstrated effective in previous work of others and our own (Boublil *et al* 2015, Yang *et al* 2018, Wang *et al* 2020).

The chosen input and label patches from ${\boldsymbol{f}}$ and ${\boldsymbol{u}}$ form the input data matrix $\widetilde{{\boldsymbol{X}}}\in {{\mathfrak{R}}}^{64\times K}$ and the label matrix ${\boldsymbol{X}}\in {{\mathfrak{R}}}^{8\times K}$ with *K* = 100 000. To equally distribute importance of each row in the input matrix, we scale the intensities of each row to the range of [−1, 1] using min-max scaling ${\widetilde{x}}_{{\mathrm{scaled}}}=2(\widetilde{x}-{\widetilde{x}}_{\min })/({\widetilde{x}}_{\max }-{\widetilde{x}}_{\min })-1,$ where $\widetilde{x}$ is an element in a specific row of $\widetilde{{\boldsymbol{X}}},$ and ${\widetilde{x}}_{\min }$ and ${\widetilde{x}}_{\max }$ denote the minimum and maximum of that row. Each row in the label matrix ${\boldsymbol{X}}$ is scaled similarly. The scaled input matrix ${\widetilde{{\boldsymbol{X}}}}_{{\mathrm{scaled}}}$ and label matrix ${{\boldsymbol{X}}}_{{\mathrm{scaled}}}$ constitute the training data.

#### 2.1.2. Model training

As shown in figure 1(a), the ANN network is used to represent the mapping function $\widetilde{F}.$ The input and label layers directly map to the generated input and label column vectors in ${\widetilde{{\boldsymbol{X}}}}_{{\mathrm{scaled}}}$ and ${{\boldsymbol{X}}}_{{\mathrm{scaled}}}.$ Therefore, the number of neurons in the input ${n}_{{\mathrm{inp}}}$ and label/output layers ${n}_{{\mathrm{lab}}}$/${n}_{{\mathrm{out}}}$ are 64 and 8, respectively. We set the number of neurons in the hidden layer ${n}_{{\mathrm{hid}}}$ to 128 empirically.

Given a training input ${\widetilde{{\boldsymbol{x}}}}_{{\mathrm{scaled}}},$ the deterministic mapping ${h}_{{{\boldsymbol{\theta }}}_{{\bf{1}}}}$ that transforms ${\widetilde{{\boldsymbol{x}}}}_{{\mathrm{scaled}}}$ into a hidden representation ${\boldsymbol{\alpha }}:$

\begin{eqnarray*}{\boldsymbol{\alpha }}={h}_{{{\boldsymbol{\theta }}}_{{\bf{1}}}}\left({\widetilde{{\boldsymbol{x}}}}_{{\mathrm{scaled}}}\right)={\mathrm{ReLU}}\left({{\boldsymbol{W}}}_{{\bf{1}}}{\widetilde{{\boldsymbol{x}}}}_{{\mathrm{scaled}}}+{{\boldsymbol{b}}}_{{\bf{1}}}\right),\end{eqnarray*}

where ${{\boldsymbol{\theta }}}_{{\bf{1}}}=\{{{\boldsymbol{W}}}_{{\bf{1}}},{{\boldsymbol{b}}}_{{\bf{1}}}\}$ is the first parameter set with ${{\boldsymbol{W}}}_{{\bf{1}}}\in {{\mathfrak{R}}}^{{n}_{\mathrm{hid}}\times {n}_{\mathrm{inp}}}$ as a weighting matrix consisting of weights assigned to the connections between neurons and ${{\boldsymbol{b}}}_{{\bf{1}}}\in {{\mathfrak{R}}}^{{n}_{\mathrm{hid}}\times 1}$ as an offset vector. The rectified linear unit (ReLU) (Glorot *et al* 2011) is used as the nonlinear activation function. The resulting hidden representation ${\boldsymbol{\alpha }}$ is then mapped back to a recovered vector:

\begin{eqnarray*}{\boldsymbol{\beta }}={g}_{{{\boldsymbol{\theta }}}_{{\bf{2}}}}\left({\boldsymbol{\alpha }}\right)={{\boldsymbol{W}}}_{{\bf{2}}}{\boldsymbol{\alpha }}+{{\boldsymbol{b}}}_{{\bf{2}}},\end{eqnarray*}

where the second parameter set ${{\boldsymbol{\theta }}}_{{\bf{2}}}=\left\{{{\boldsymbol{W}}}_{{\bf{2}}}\in {{\mathfrak{R}}}^{{n}_{{\mathrm{out}}}\times {n}_{{\mathrm{hid}}}},{{\boldsymbol{b}}}_{{\bf{2}}}\in {{\mathfrak{R}}}^{{n}_{{\mathrm{out}}}\times 1}\right\}.$

Training the mapping function $\widetilde{F}$ with ${\widetilde{{\boldsymbol{X}}}}_{{\mathrm{scaled}}}$ and ${{\boldsymbol{X}}}_{{\mathrm{scaled}}}$ therefore becomes learning the parameters of ${{\boldsymbol{\theta }}}_{{\bf{1}}}$ and ${{\boldsymbol{\theta }}}_{{\bf{2}}}$ through minimizing the recovery loss averaged among all the training input vectors:

\begin{eqnarray*}{\mathrm{\arg }}\mathop{\min }\limits_{{{\boldsymbol{\theta }}}_{{\bf{1}}},\,{{\boldsymbol{\theta }}}_{{\bf{2}}}}\frac{1}{K}\displaystyle \sum _{k=1}^{K}{\unicode{x02016}{{\boldsymbol{\beta }}}^{k}-{{\boldsymbol{x}}}_{{\mathrm{scaled}}}^{k}\unicode{x02016}}^{2},\end{eqnarray*}

where ${{\boldsymbol{\beta }}}^{k}=\widetilde{F}\left({{\boldsymbol{\theta }}}_{{\bf{1}}},{{\boldsymbol{\theta }}}_{{\bf{2}}},{\widetilde{{\boldsymbol{x}}}}_{\mathrm{scaled}}^{k}\right).$ We apply the classic backpropagation algorithm, stochastic gradient descent (SGD) (LeCun *et al* 2015), to solve this nonlinear regression problem. This will result in learned feature detectors described by ${{\boldsymbol{W}}}_{{\bf{1}}}$ and ${{\boldsymbol{b}}}_{{\bf{1}}}$ being able to separate useful information from noise for each training input ${\widetilde{{\boldsymbol{x}}}}_{{\mathrm{scaled}}}^{k}.$ The SGD algorithm is implemented with the learning rate initialized as 0.01 and decreased during training based on the inverse decay policy (Jia *et al* 2014). The total iteration number of SGD is set to 150 000 empirically to ensure convergence (see figure S1 for the convergence behaviour).

#### 2.1.3. Testing data processing

After the model is trained, we apply the ANN model to denoise the dynamic frames of testing subjects reconstructed from their 1/10-count data. For each location in a testing image ${\boldsymbol{z}},$ a testing input vector is generated by extracting the $4\times 4\times 4$ patch, removing the patch mean value, stacking the patch voxels into a column vector, and scaling each entry of the vector using the same min-max scaling factors as in the training input data to reinforce the generalization from training patches to the unlearned testing patches. The generated testing input vector is then fed to the trained ANN model to compute a recovery vector of size $8\times 1$ as the network output, which corresponds to the central section of $2\times 2\times 2$ of the testing patch before the ANN processing. We scale back the recovery vector using the maximum and minimum of each row in the training label data, add back the mean of corresponding testing patch to the vector, and arrange the vector back to form a recovery patch. Since each voxel in the denoised version of ${\boldsymbol{z}}$ is covered by 8 patches, the final voxel estimate is calculated by averaging these contributions. Compared with outputting a patch with the same size as the input patch, which means that the final voxel estimate will be from averaging over more contributions, our method with smaller output patch is expected to perform better in keeping the regional mean.

We adopt a cascade strategy to further make use of the denoising capability of the ANN model, applying it multiple times in the whole process (figure 1(a)). The output of the ANN model described above will go through the input generation step before being fed into the model again for another round of denoising. As a denoising technique, the noise reduction performance of the trained ANN model can be tuned by applying it different number of times within the cascade strategy during the testing phase. Taking the denoising procedure-introduced bias into consideration, we obtain the final result after applying the model three times in this study. More discussion about the number of applications will be provided.

### 2.2. ANN+HYPR spatiotemporal denoising

We adopt the HYPR strategy to further process the ANN denoised image series to make use of entire data acquired during dynamic imaging, with the procedure diagram shown in figure 1(b). The HYPR technique, which is easy to implement and fast to accomplish, has been demonstrated successful in denoising dynamic MRI and PET image sequences while preserving spatial resolution. It involves generating a composite image from all or part of the time series, which provides better spatial resolution, and calculating weighting matrices from individual time frames to maintain temporal resolution. We use all image frames in the dynamic imaging procedure to create the time-averaged higher SNR composite image based on the duration of each time frame. For each frame, the weighting image is computed as the ratio of the box-filtered individual image and the box-filtered composite image. The HYPR processed image frames then come from multiplying each weighting image with the composite image.

### 2.3. Methods used for comparison

To assess the performance of the proposed ANN+HYPR method, we implemented the following approaches for comparison: Gaussian filtering, the original HYPR processing, and the spatiotemporal NLM (NLM-ST) algorithm. Gaussian filtering is the most commonly applied denoising method in clinical practice. We choose a set of full width at half maximum (FWHM) values for 3D Gaussian filtering to reach the comparable noise reduction to what the ANN model achieves after being applied each time. This way we can compare the ANN model with Gaussian filtering in terms of resolution preservation while reducing similar noise. The original HYPR processing with different box filter sizes is implemented to process the reduced-dose image frames so that the effect of ANN individual frame denoising before the HYPR strategy application could be appreciated.

We also implemented the NLM-ST algorithm as an integrated spatiotemporal denoising approach (Dutta *et al* 2013) to compare with the proposed ANN+HYPR method in their performance in reduced-dose dynamic imaging on both individual frames and parametric images after kinetic analysis. Based on the original work, we used 7 image frames (9th–15th frames) in the temporal dimension to constitute the search windows (11 × 11 × 11 × 7) and neighborhood windows (7 × 7 × 7 × 7). Note that for a fair comparison, we advanced the 2D spatial windows employed in the original work to 3D. A set of smoothing parameters in similarity weight calculation were chosen for the NLM-ST method to match noise reduction of the proposed method for reasonable comparisons.

## 3. Experiments

In this study, we trained the ANN with reduced-dose and full-dose dynamic image frame pairs of a HC subject and applied the trained model to reduced-dose image frames of ten other subjects. We evaluated the proposed ANN+HYPR method and other denoising methods in terms of noise versus bias tradeoff of the processed dynamic frames and the parametric images estimated from them.

### 3.1. Patient data acquisition

Six HC subjects (four males and two females; age 52 ± 19 years; weight 87 ± 16 kg) and five PD patients (two males and three females; age 63 ± 12 years; weight 71 ± 6 kg) were included in this study with one randomly chosen HC subject to train the ANN and all others for evaluation. These data were acquired in a previous study (Matuskey *et al* 2020) under a protocol approved by the Yale University Human Investigation Committee and the Yale New Haven Hospital Radiation Safety Committee, in accordance with the U.S. federal policy for the protection of human research subjects contained in Title 45 Part 46 of the Code of Federal Regulations (45 CFR 46). Written informed consent was collected from each of the subjects.

All subjects underwent arterial cannulation and blood was collected for measurement of the time course of ^11^C-UCB-J in plasma, including radiometabolite analysis. Every subject had both an MRI scan and a dynamic PET scan, with the T1-weighted MRI performed on a 3T Trio scanner (Siemens Medical Solution, Erlangen, Germany) with a circularly polarized head coil. The dynamic ^11^C-UCB-J PET scans were performed on a High-Resolution Research Tomograph (Siemens/CTI, Knoxville, TN), which resulted in reconstructed images of 207 slices (1.2 mm slice separation) with image resolution (FWHM) of ∼3 mm. The injected mass was limited to 10 *μ*g of ^11^C-UCB-J and the injected dose of ^11^C-UCB-J was 510 ± 212 MBq for HCs and 575 ± 234 MBq for PD patients, respectively. Before every ^11^C-UCB-J injection, a 6 min transmission scan was performed for attenuation correction.

The PET data were acquired in list mode for 60 min after the start of the ^11^C-UCB-J administration. We sub-sampled the original, i.e. full-count dataset with a ratio of 1/10 by redistributing events in the list mode files sequentially to 10 subsets, resulting in 10 noise realizations of 1/10-count datasets. The time step of event redistribution was set as 1 millisecond to minimize the total count variation among the 10 noise realizations. The full-count and the 1/10-count data were then binned into 21 sinograms with the following frame durations: 6 × 30 s, 3 × 1 min, 2 × 2 min, and 10 × 5 min.

### 3.2. Image reconstruction and kinetic analysis

For each subject, the dynamic emission data with full counts or 1/10 counts were reconstructed into 21 frames with correction for attenuation, normalization, scatter, randoms, and dead time by the MOLAR algorithm (Carson *et al* 2003). Event-by-event motion correction (Jin *et al* 2013) was included in the reconstruction based on motion detection with a Polaris Vicra optical tracking system (NDI systems, Waterloo, Ontario, Canada) using reflectors mounted on a swim cap worn by the subject. To further eliminate any residual motion, the reconstructed dynamic frames were coregistered to an early summed PET image (0–10 min after the ^11^C-UCB-J injection) using a six-parameter mutual information algorithm (FMRIB’s Linear Image Registration Tool, FMRIB Software Library).

Among the reconstructed image frames, the 12th frame (10–15 min after injection) has the most counts and therefore the least noise. One noise realization of the 1/10-count image of this frame and its corresponding full-count image of the training HC was used to train the ANN model. Considerations for training image selection will be presented in Discussion.

The one-tissue (1T) compartment model has been demonstrated reliable to estimate the parametric images in human studies of SV2A PET imaging using ^11^C-UCB-J (Koole *et al* 2019, Mansur *et al* 2020). Therefore, given the full-count and the 1/10-count dynamic frames with or without denoising, we performed kinetic analysis voxel by voxel using the 1T compartment model with the metabolite-corrected arterial plasma curve to generate the parametric images of the tracer uptake *K*_1_ and distribution volume *V*_T_. Data points were weighted on the basis of noise equivalent counts in each frame (Pajevic *et al* 1998, Finnema *et al* 2018). We applied a basis function method with the washout rate *k*_2_ limited to the range of 0.01–1.0 min^−1^ for fitting the *K*_1_ and *k*_2_, and *V*_T_ was calculated as *K*_1_/*k*_2_.

### 3.3. Performance evaluation

For the subjects used in this study, the early summed motion corrected PET image was registered to the subject’s MR image (Mecca *et al* 2020). Cortical reconstruction and volumetric segmentation were performed for each subject’s MR image using FreeSurfer (Fischl 2012). The following 13 structural regions of interest (ROIs) from the FreeSurfer segmentation were used in this study: amygdala, caudate nucleus, cerebellum, anterior cingulum gyrus, posterior cingulum gyrus, frontal cortex, hippocampus, insular cortex, occipital cortex, parietal cortex, putamen, temporal cortex, and thalamus.

To quantitatively evaluate the voxel-level noise in the reconstructed activity image frames and the thereafter estimated parametric images from the 1/10-count with and without processing, we calculated the ensemble normalized standard deviation (EnNSD) across 10 noise realizations as a measure of noise:

\begin{eqnarray*}{\mathrm{EnNSD}}=\frac{1}{m}\displaystyle \sum _{i=1}^{m}\frac{\sqrt{\frac{1}{9}\displaystyle {\sum }_{j=1}^{10}{({f}_{1/10,i}^{j}-{\mathop{f}\limits^\unicode{x00305}}_{1/10,i})}^{2}}}{{\mathop{f}\limits^\unicode{x00305}}_{1/10,i}},\end{eqnarray*}

where ${f}_{1/10,i}^{j}$ denotes the $i$th voxel in each ROI of a dynamic frame or a parametric image at the $j$ th noise realization of 1/10 counts, ${\mathop{f}\limits^\unicode{x00305}}_{1/10,i}=\frac{1}{10}\displaystyle {\sum }_{j=1}^{10}{f}_{1/10,i}^{j}$ is the mean value of the $i$th voxel across 10 noise realizations, and $m$ is the number of voxels in the ROI. The noise reduction (NR) achieved by a denoising method with respect to the noise in the 1/10-count images is defined as:

\begin{eqnarray*}{\mathrm{NR}}=\frac{{{\mathrm{EnNSD}}}_{1/10}-{{\mathrm{EnNSD}}}_{1/10{\mathrm{processed}}}}{{{\mathrm{EnNSD}}}_{1/10}}\times 100 \% .\end{eqnarray*}

The mean value within a specified structural ROI has been used to measure the synaptic density in dynamic SV2A PET imaging (Bastin *et al* 2020, Wilson *et al* 2020, Naganawa *et al* 2021, Salmon *et al* 2021). Our goal is to assess the effect of each denoising technique on the quantitative measurement of synaptic density after processing the 1/10-count images. To compare the regional difference between the 1/10-count dynamic frames or parametric images with those estimated from the full-count counterparts, the relative error (RE) of the regional mean value as a measure of bias was calculated for each region:

\begin{eqnarray*}{\mathrm{RE}}=\frac{1}{10}\displaystyle \sum _{j=1}^{10}\frac{{\mathop{f}\limits^\unicode{x00305}}_{1/10}^{j}-{\mathop{f}\limits^\unicode{x00305}}_{{\mathrm{full}}}}{{\mathop{f}\limits^\unicode{x00305}}_{{\mathrm{full}}}},\end{eqnarray*}

where ${\mathop{f}\limits^\unicode{x00305}}_{1/10}^{j}=\frac{1}{m}\displaystyle {\sum }_{i=1}^{m}{f}_{1/10,i}^{j}$ is the regional mean intensity value of the dynamic frame or the parametric image of the $j$ th noise realization, and ${\mathop{f}\limits^\unicode{x00305}}_{{\mathrm{full}}}=\frac{1}{m}\displaystyle {\sum }_{i=1}^{m}{f}_{{\mathrm{full}},i}$ denotes the regional mean intensity value from the full-count data. $i$ and $m$ are defined as above.

For each method, we plotted the noise (EnNSD) against bias (RE) along with the number of ANN model applications or increased smoothing parameters on all the ROIs in the dynamic frames or the parametric images of every testing subject for a fair comparison of the denoising capability.

## 4. Results

### 4.1. Noise reduction in dynamic frames

**Figure 2.**
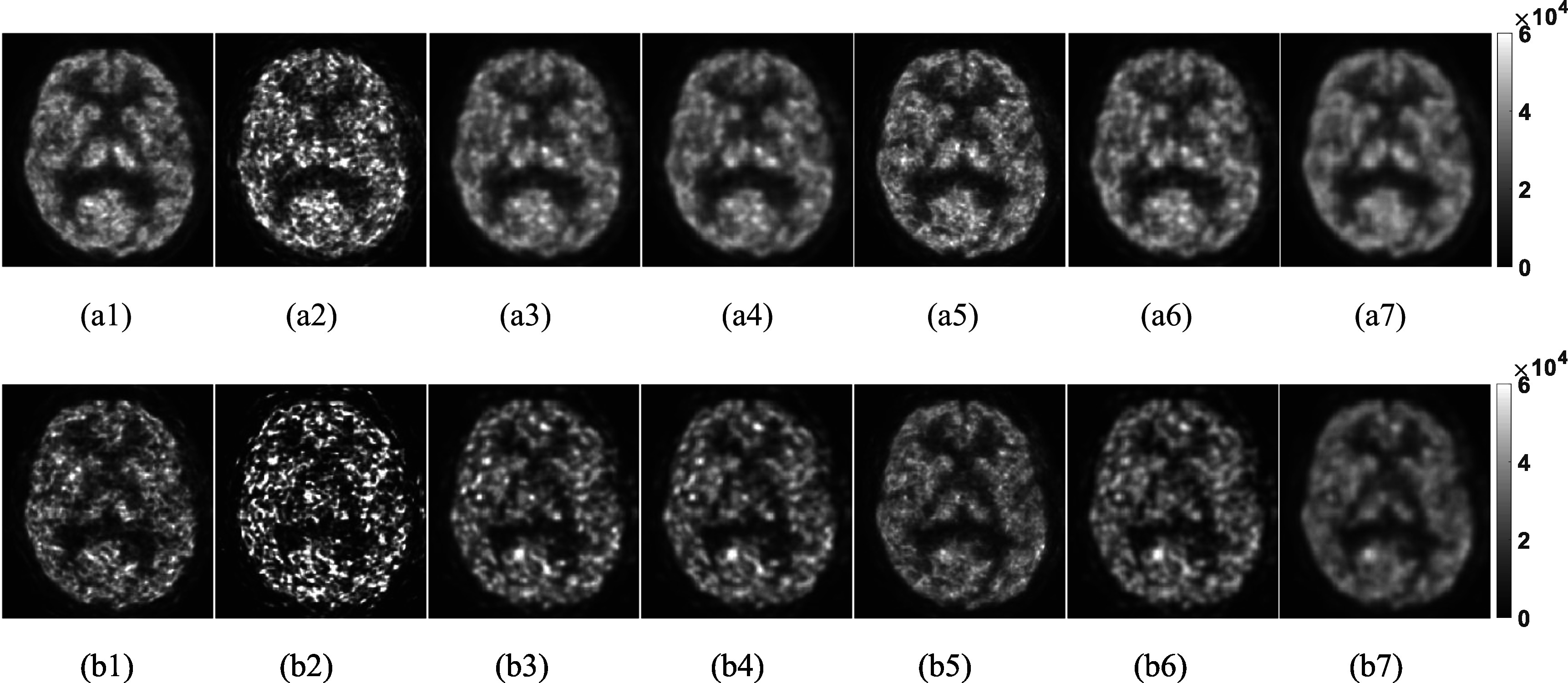
Transaxial slices (Bq/mL) through the (a) 12th and (b) 21st frames reconstructed from (1) full-count data, (2) 1/10-count data, and after (3) 3D Gaussian (FWHM = 3.1 mm), (4) NLM-ST (the smoothing parameter = 1.5), (5) HYPR (the 7 × 7 × 7 box filter), (6) the cascade ANN (applied three times), and (7) the ANN+HYPR (three times + the 7 × 7 × 7 box filter) processing. Data shown come from a PD patient.

We first show the results of a PD patient starting from the activity image frames. One noise realization of two representative frames, an earlier and a later one (12th, 10 to 15 min and 21st, 55 to 60 min after injection), reconstructed from full counts and 1/10 counts pre- and post-denoising by different methods are displayed in figure 2. The 12th frame has lower noise while the 21st frame at the end of acquisition has higher noise. The Gaussian filtered images shown were obtained using a 3D filter with the FWHM set as 3.1 mm to match the noise level in the ANN denoised images by applying the trained model three times. The HYPR denoised images came from using the box filter of 7 × 7 × 7 voxels while the NLM-ST denoised images were generated using a global smoothing constant of 1.5. In the last column, we show the ANN+HYPR processed images with ANN processed three times and the 7 × 7 × 7 box filter in HYPR. Compared with the raw 1/10-count images, the obvious noise reduction achieved by different processing methods can be observed. The quantitative evaluation to measure the noise and bias of the denoised frames is presented next.

**Figure 3.**
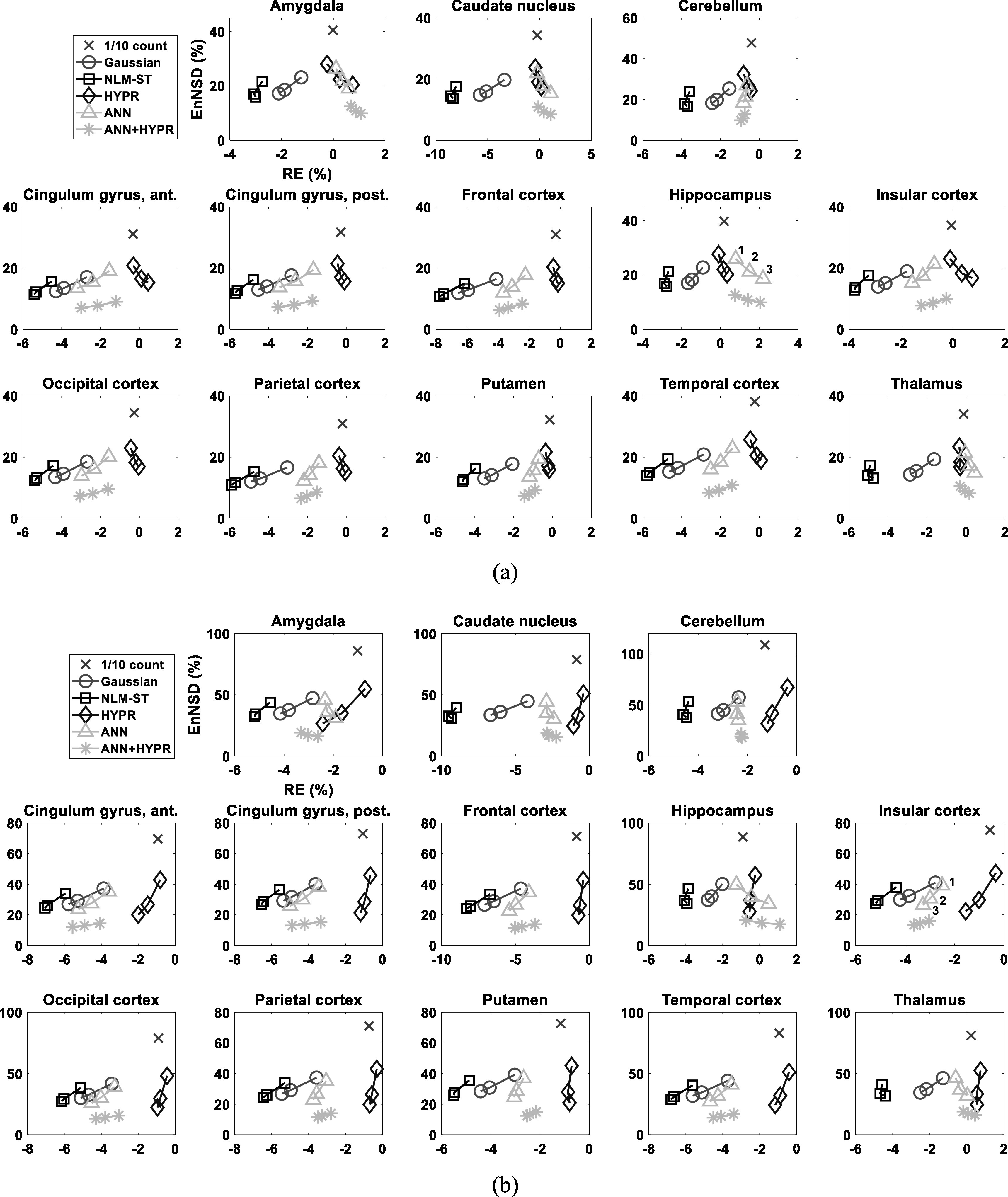
Ensemble normalized standard deviation (EnNSD) versus relative error (RE) plots of the 1/10 count dynamic frames, frames after applying 3D Gaussian (FWHM = 2.2, 2.8, 3.1 mm), NLM-ST (the smoothing parameter = 0.5, 1.0, 1.5), HYPR (the box filter size = 3 × 3 × 3, 5 × 5 × 5, and 7 × 7 × 7), the cascade ANN (once, twice, and three times), and the ANN+HYPR (once, twice, and three times + the 7 × 7 × 7 box filter) processing for (a) the 12th frame and (b) the 21st frame, averaged over individual ROIs of 10 testing subjects.

Figure 3 plots the EnNSD versus RE (noise versus bias) on the 13 FreeSurfer defined ROIs averaged among the ten testing subjects. The three points for the ANN come from applying the model once, twice, and three times (indicated with ‘1’, ‘2’, and ‘3’ in figure 3), respectively. The evaluation plots for ANN+HYPR are obtained from applying the HYPR algorithm with the box filter size of 7 × 7 × 7 to each of the ANN results. Multiple applications of the ANN model reduce the noise more while slightly increasing the bias. Designed to achieve the similar noise reduction as ANN processing once, twice, and three times, the Gaussian filtering (FWHM = 2.2, 2.8, 3.1 mm) introduces larger bias in all ROIs. The NLM-ST (the smoothing parameter = 0.5, 1.0, 1.5) does not perform as well as Gaussian in terms of noise versus bias tradeoff probably due to small contrast between the ROIs and their neighborhood regions, as also shown in similar studies (Dutta *et al* 2013, Chan *et al* 2014). Making use of information from the entire time series, the original HYPR algorithm (with box filter sizes of 3 × 3 × 3, 5 × 5 × 5, and 7 × 7 × 7 plotted) reduces noise while introducing less bias compared with other denoising methods, especially for frame 21. The minor uptake pattern changes among the dynamic ^11^C-UCB-J frames during the dynamic scan certainly contribute to the success of HYPR processing. Compared with the spatial-only ANN processing, ANN+HYPR (the 7 × 7 × 7 box filter) reduces noise more without introducing noteworthy bias. In addition, ANN+HYPR reduces appreciably more noise than HYPR alone through box filter size adjustment while introducing minimal bias. The EnNSD and RE averaged over 13 ROIs for all image frames besides the 12th and the 21st of a testing subject are shown in figure S2.

**Figure 4.**
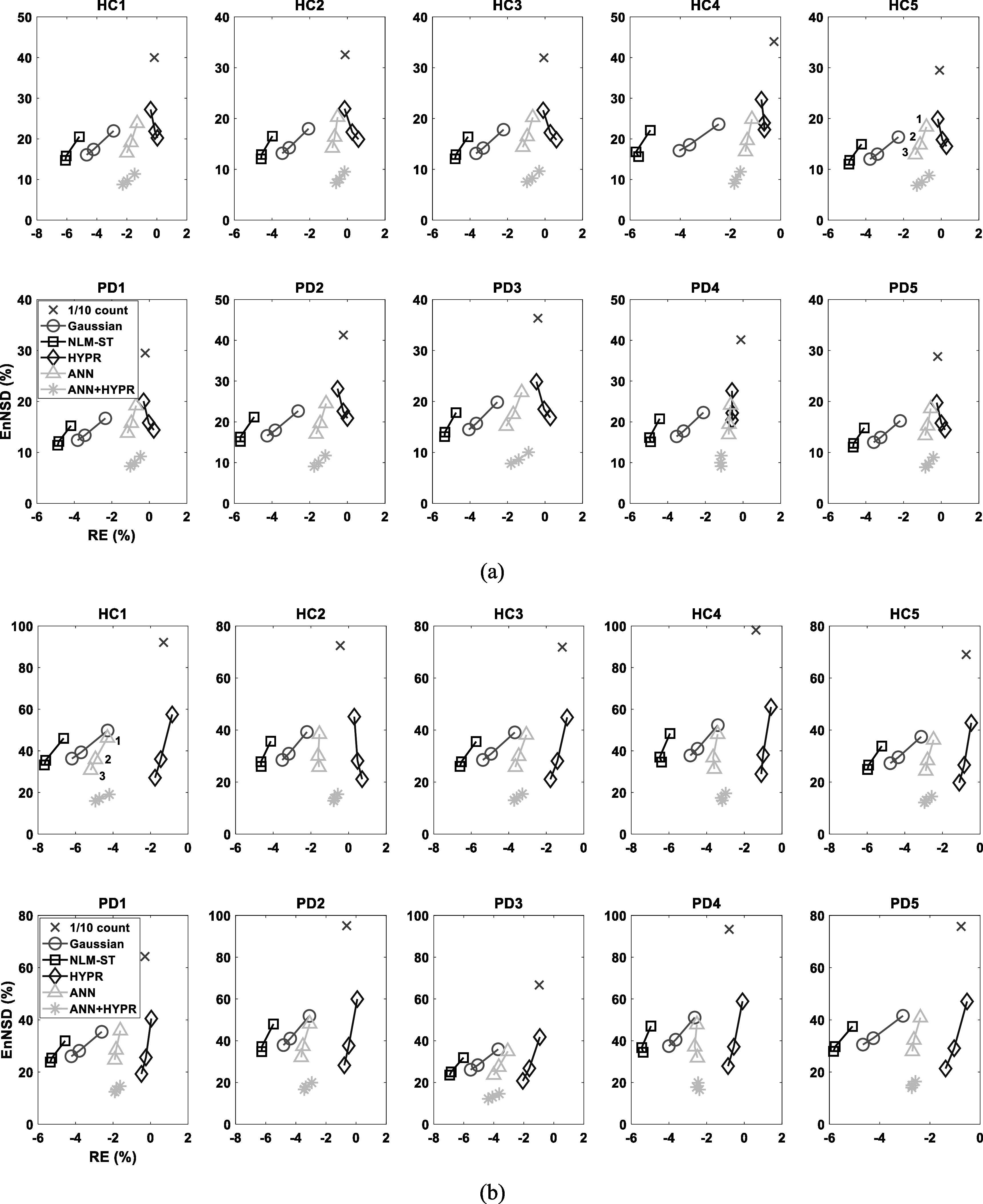
Ensemble normalized standard deviation (EnNSD) versus relative error (RE) plots of the 1/10 count dynamic frames, frames after applying 3D Gaussian (FWHM = 2.2, 2.8, 3.1 mm), NLM-ST (the smoothing parameter = 0.5, 1.0, 1.5), HYPR (the box filter size = 3 × 3 × 3, 5 × 5 × 5, and 7 × 7 × 7), the cascade ANN (once, twice, and three times), and the ANN+HYPR (once, twice, and three times + the 7 × 7 × 7 box filter) processing for (a) the 12th frame and (b) the 21st frame, averaged over 13 ROIs of each testing subject.

To pool results across regions for each testing subject, we averaged the EnNSD and RE calculated on individual ROIs and present them in figure 4. The EnNSD, RE, and NR averaged over regions and frames of all subjects with the parameter specifications as those presented in figure 2 are also summarized in table 1. Similar conclusions with regard to the different denoising methods can be drawn to those provided for figure 3.

**Table 1.** Quantitative evaluation results of different methods, Gaussian (FWHM = 3.1 mm), NLM-ST (the smoothing parameter = 1.5), HYPR (the 7 × 7 × 7 box filter), the ANN (applied three times), and the ANN + HYPR (three times + the 7 × 7 × 7 box filter), on dynamic frames averaged over 10 subjects. (RE: relative error, EnNSD: ensemble normalized standard deviation, and NR: noise reduction. All data are shown in percentage.).

Methods		1/10	Gaussian	NLM-ST	HYPR	ANN	ANN+HYPR
Frame 12	RE	−0.2	−3.9	−5.2	0.1	−1.3	−1.4
	EnNSD	35.4	14.3	13.1	17.6	15.1	8.0
	NR		59.4	62.8	50.4	57.0	77.3
Frame 21	RE	−0.8	−4.8	−6.1	−1.1	−3.2	−3.0
	EnNSD	79.9	31.6	28.9	23.5	27.8	14.2
	NR		60.4	63.8	70.5	65.0	82.1
All frames	RE	−0.8	−4.3	−5.5	−0.2	−2.6	−2.0
	EnNSD	76.9	32.7	30.5	25.4	29.0	15.0
	NR		57.6	60.5	67.1	62.1	80.5

### 4.2. Noise reduction in parametric images

**Figure 5.**
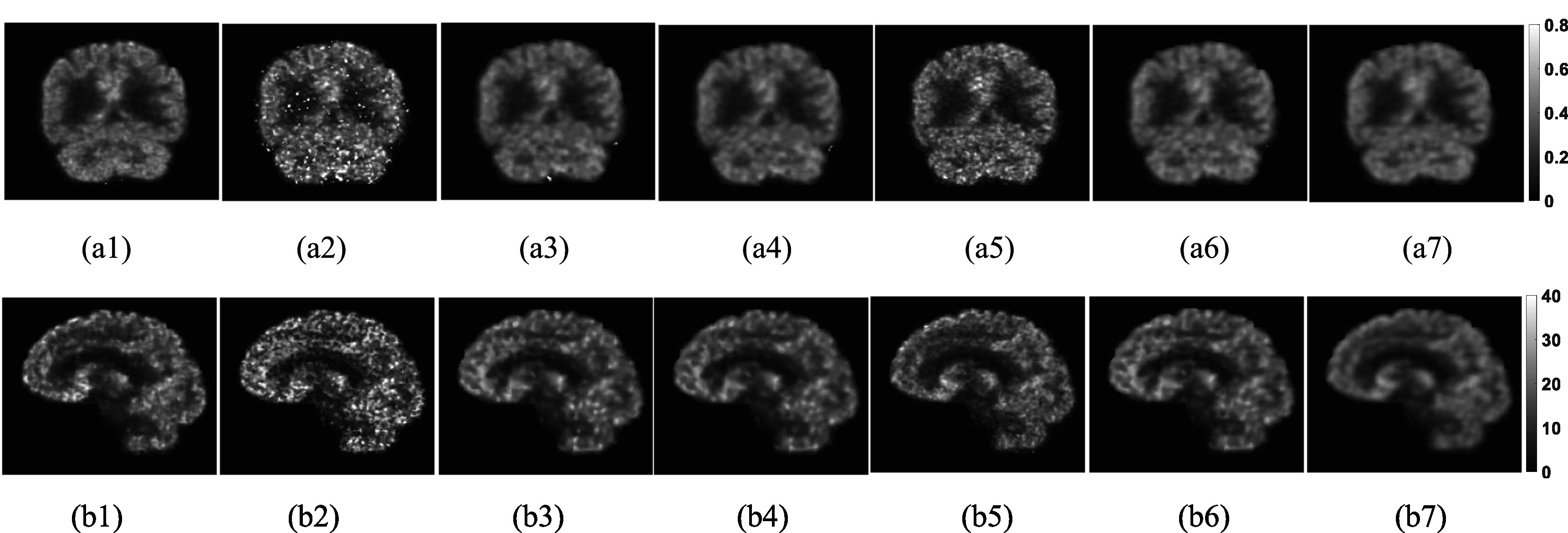
Coronal slices of (a) *K*_1_ and (b) sagittal slices of *V*_T_ derived from kinetic analysis estimated from the (1) full-count dynamic frames, (2) 1/10-count dynamic frames, (3) 3D Gaussian (FWHM = 3.1 mm), (4) NLM-ST (the smoothing parameter = 1.5), (5) HYPR (the 7 × 7 × 7 box filter), (6) the cascade ANN (applied three times), and (7) the ANN+HYPR (three times + the 7 × 7 × 7 box filter) processed 1/10-count dynamic frames. Data shown come from a PD patient.

Figure 5 shows coronal slices of *K*_1_ and sagittal slices of *V*_T_ images of the same PD patient as presented in figure 2, generated from 1/10-count dynamic frames processed by different denoising methods. The noticeable high noise in the inferior portion, especially in the coronal slices is due to the head position being close to the axial edge of the scanner, which has lower sensitivity. Reducing counts to generate dynamic image frames leads to noisier parametric images as compared with the full-count results, while processing the dynamic frames by the proposed ANN+HYPR method and other denoising methods obviously lowers the noise. Although the original HYPR alone performs very well in dynamic frame denoising, its advantage does not fully translate into the parametric images. Noisier *K*_1_ and *V*_T_ images from HYPR can be seen while quantitative evaluation below further reveals the details.

**Figure 6.**
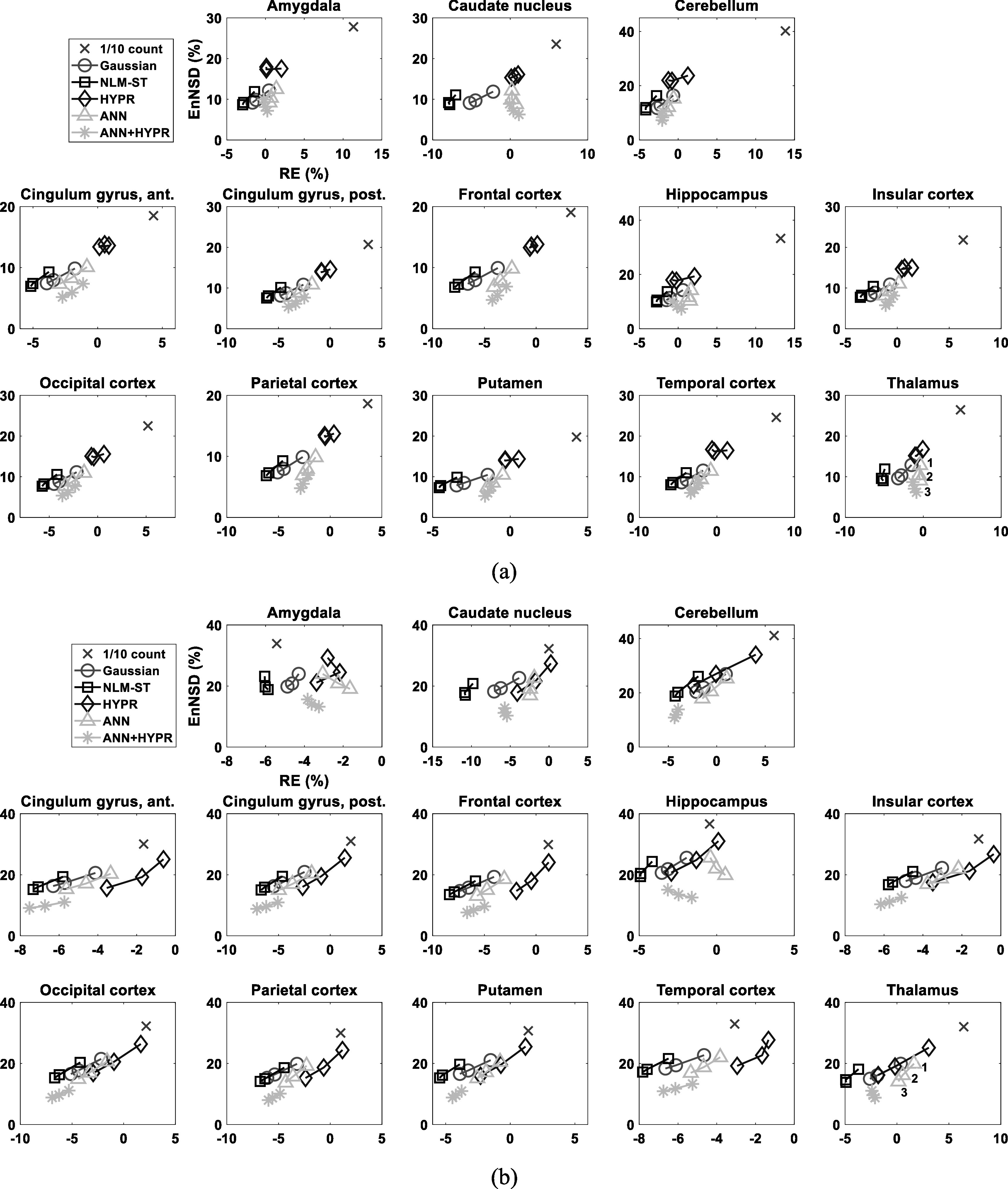
Ensemble normalized standard deviation (EnNSD) versus relative error (RE) plots of (a) *K*_1_ images and (b) *V*_T_ images estimated from the 1/10 count dynamic frames, those estimated from 3D Gaussian (FWHM = 2.2, 2.8, 3.1 mm), NLM-ST (the smoothing parameter = 0.5, 1.0, 1.5), HYPR (the box filter size = 3 × 3 × 3, 5 × 5 × 5, and 7 × 7 × 7), the cascade ANN (once, twice, and three times), and the ANN+HYPR (once, twice, and three times + the 7 × 7 × 7 box filter) denoised 1/10 count dynamic frames, averaged over individual ROIs of 10 testing subjects.

**Figure 7.**
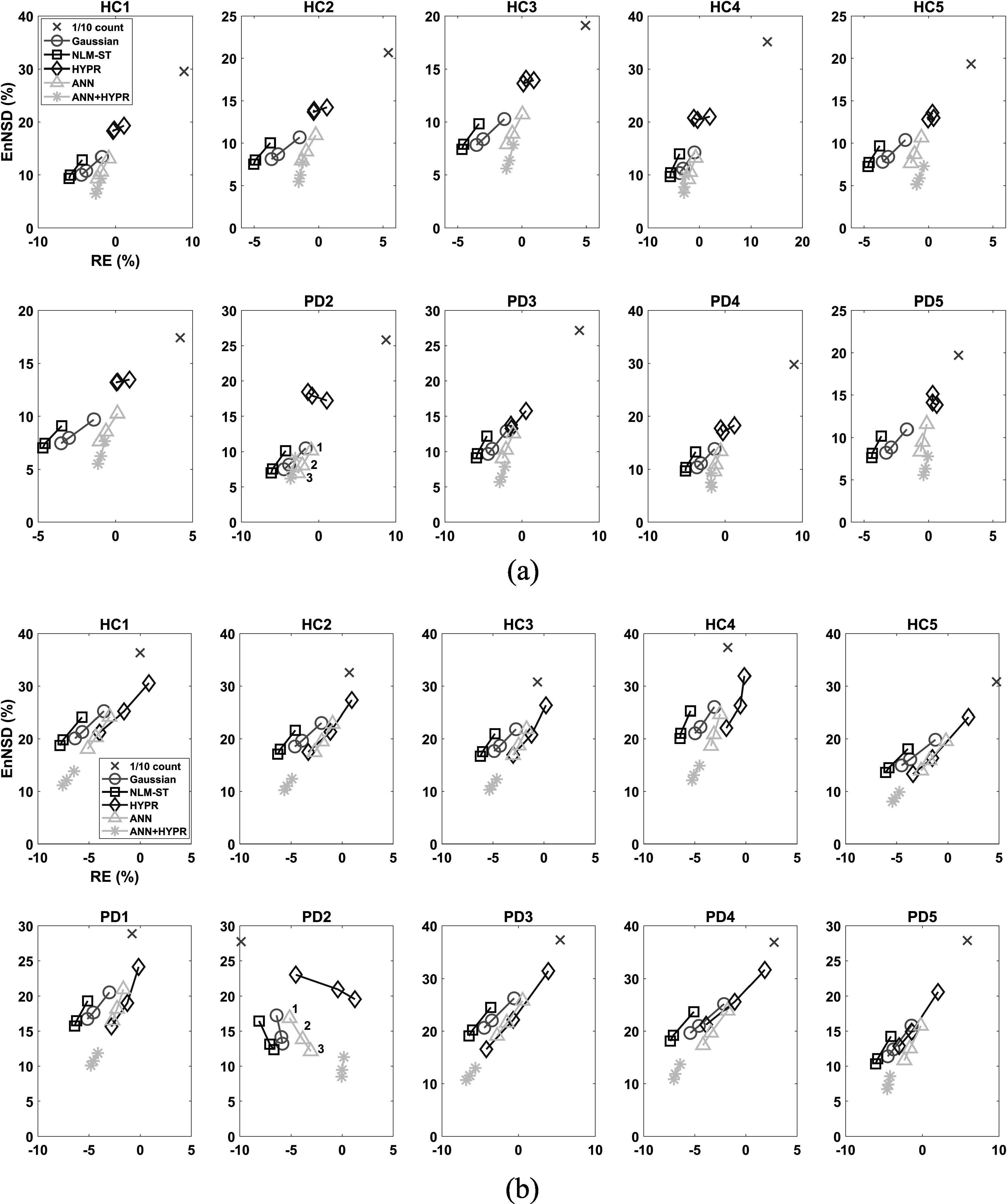
Ensemble normalized standard deviation (EnNSD) versus relative error (RE) plots of (a) *K*_1_ images and (b) *V*_T_ images estimated from the 1/10 count dynamic frames, those estimated from 3D Gaussian (FWHM = 2.2, 2.8, 3.1 mm), NLM-ST (the smoothing parameter = 0.5, 1.0, 1.5), HYPR (the box filter size = 3 × 3 × 3, 5 × 5 × 5, and 7 × 7 × 7), the cascade ANN (once, twice, and three times), and the ANN+HYPR (once, twice, and three times + the 7 × 7 × 7 box filter) denoised 1/10 count dynamic frames, averaged over 13 ROIs of each testing subject.

The EnNSD versus RE plots for the parametric images of individual ROIs averaged over ten subjects are shown in figure 6 (similar format to figure 3). The advantage of the ANN spatial denoising over 3D Gaussian filtering can be appreciated in the reduced bias in most of the ROIs. Although HYPR processing performs well in terms of noise versus bias tradeoff of individual frames (as shown in figures 3 and 4), its performance in the estimated parametric images after kinetic analysis, especially in reducing noise in *K*_1_ appears less effective than other methods. This is probably because noise reduction that could be accomplished in kinetic analysis has been partially redeemed by HYPR, which serves as a temporal filter using the time-averaged composite image to calculate the processed activity frames. The total noise reduction after curve fitting to obtain parametric images is therefore not as significant as other methods. Compared with HYPR alone and the ANN spatial processing, ANN+HYPR further reduces noise while introducing minimal bias in *K*_1_ or *V*_T_. In addition to the results averaged over 10 subjects for individual ROIs, we also show the results averaged over the 13 ROIs of each subject in figure 7. Similar conclusions to that from figure 6 can be drawn regarding the relative performance among the results from different denoising methods. Evaluations for the parametric images of each subject on individual ROIs are shown in figures S3 and S4. The EnNSD, RE, and corresponding NR of *K*_1_ and *V*_T_ images averaged over all subjects are listed in table 2. These results confirm the advantage of the ANN+HYPR denoising over other methods by reaching more noise reduction while introducing reasonably low bias.

**Table 2.** Quantitative evaluation results of different methods, Gaussian (FWHM = 3.1 mm), NLM-ST (the smoothing parameter = 1.5), HYPR (the 7 × 7 × 7 box filter), the ANN (applied three times), and the ANN + HYPR (three times + the 7 × 7 × 7 box filter), on the parametric images (*K*_1_ and *V*_T_) averaged over 10 subjects. (RE: relative error, EnNSD: ensemble normalized standard deviation, and NR: noise reduction. All data are shown in percentage.).

Methods		1/10	Gaussian	NLM-ST	HYPR	ANN	ANN+HYPR
*K*_1_	RE	6.7	−3.8	−5.2	−0.4	−1.7	−1.9
	EnNSD	24.4	8.7	8.2	15.7	8.3	5.9
	NR		63.2	69.5	34.2	64.5	75.0
*V*_T_	RE	0.6	−5.1	−6.6	−2.8	−3.2	−5.3
	EnNSD	32.7	17.4	16.2	17.7	16.1	9.9
	NR		47.1	50.8	45.8	50.9	69.8

## 5. Discussion

In this study, we proposed an ANN+HYPR framework to suppress noise in dynamic frames reconstructed from reduced-dose PET data and quantitatively assessed its performance in parametric image estimation. The advantage of the proposed method over the other methods for comparison has been reflected in lowering noise in the reduced-dose parametric images more while keeping their regional bias acceptably low.

### 5.1. Noise reduction contribution

Overall, the proposed ANN+HYPR method achieves ∼80% noise reduction averaged over all dynamic frames of HC subjects and PD patients at the cost of introducing ∼2% of relative error (table 1). Although the original HYPR alone results in less bias than the proposed ANN+HYPR method in the dynamic frames, the former does not reach the noise reduction similar to what the latter does. More importantly, the proposed method brought 75% and 70% noise reduction in the resulting *K*_1_ and *V*_T_ images averaged over the ten testing subjects while introducing ∼2% and ∼5% relative error, respectively (table 2). This noise reduction is significantly larger than what the original HYPR alone (34% and 46%) and other methods achieve. Because PET data follow Poisson distribution, the noise reduction of ∼75% by the proposed ANN+HYPR method is statistically equivalent to a ∼16-fold dose increase for the *K*_1_ images, and the ∼70% noise reduction for the *V*_T_ images is statistically equivalent to an ∼11-fold dose increase. The achieved equivalent dose increase and the minimal bias introduced in the process warrant a feasible implementation of reducing the dose to 1/11 of the amount used in the current clinical practice.

### 5.2. Training data selection

**Figure 8.**
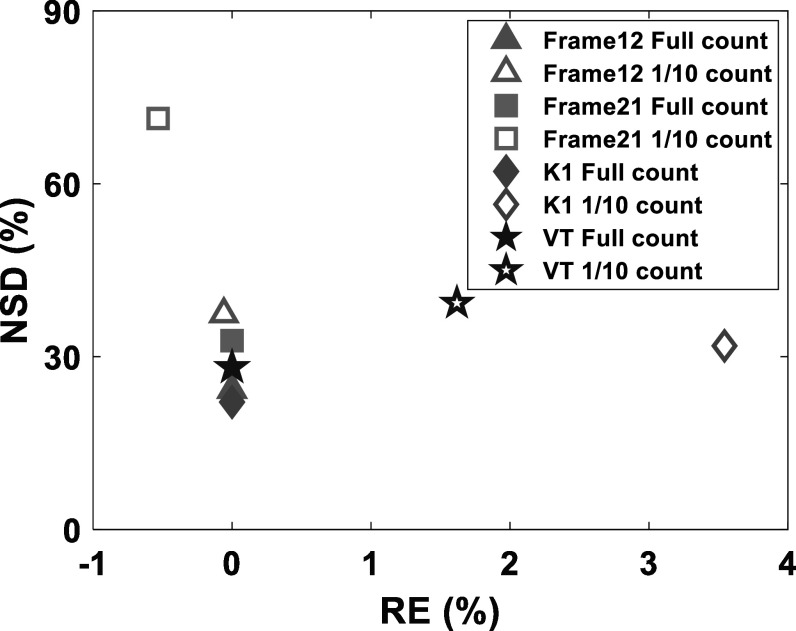
Normalized standard deviation (NSD) versus relative error (RE) plot of the 12th frame, the 21st frame, *K*_1_ image, and *V*_T_ image, averaged over 13 ROIs for the chosen training healthy control subject.

It is worth mentioning that the ANN model can be trained using any of the dynamic image frames or a combination of them. Take the 12th and 21st frames as examples. The 12th frame has the least noise among the frames and the 21st being the last one has higher noise. The regional NSD (among voxels in an ROI) versus RE averaged on the 13 ROIs of frames 12 and 21 of one noise realization of the training subject is plotted in figure 8. The noise level (NSD) difference between the 1/10-count and its corresponding full-count images of the 21st frame is larger than that of the 12th frame. Therefore, the model trained by patches extracted from the 21st frame is expected to achieve more noise reduction than the model trained by those extracted from the 12th frame. However, the higher noise in frame 21 increases the bias in the training input with respect to its desired output, which would result in built-in bias in the trained model and the bias would be carried over when the model is applied to testing subjects. Because of the negligible bias of the 1/10-count 12th frame with respect to its full-count correspondence which potentially lead to the preferred low bias in the denoised testing frames, it was chosen for the model training in this method. The evaluation results from using different training frames as well as the combination of frames are presented in figure S5 for interpretation. Since the information carried by the same frame in different noise realizations is similar, only one noise realization of the 12th frame was needed for the training input generation.

Whether it is feasible to train an ANN model with the reduced-count and full-count parametric image pair of the training subject and apply the trained model to directly denoise reduced-count parametric images of testing subjects is also worth a discussion. The increased noise in the reduced-count dynamic frames, as compared with the full-count frames, translates into considerable bias in the estimated parametric images after nonlinear fitting of the one-tissue compartment model. The regional NSD of the reduced-count *K*_1_ and *V*_T_ images and the RE values with respect to their full-count correspondence are also shown in figure 8. Similar to using frame 21 to train an ANN model, the bias of the reduced-count parametric images would introduce undesirable built-in bias in the trained model, which would also propagate to the testing datasets.

### 5.3. Cascade testing scheme

A unique feature of the proposed approach is the cascade operation which contributes to the noise reduction with obviously smaller bias than the matching Gaussian filtering (tables 1 and 2). This action was initially inspired by the development and usage of anisotropic diffusion to address the cost of edge blurring in image denoising by the standard scale-space filtering technique (Perona and Malik 1990). Gaussian kernels (holding the cascade property) of increasing variances were used to convolve with the original image to obtain a family of derived images in the scale-space approach introduced to take advantage of multiscale description of images (Babaud *et al* 1986). This one parameter family of derived images may be viewed equivalently as the solution of heat conduction or diffusion (Koenderink 1984). To address the issue of spatial distortion in the generated images due to the isotropic Gaussian smoothing, anisotropic diffusion was proposed to preserve edges, lines, or other details. In our study, the denoising ANN model was trained to best form the mapping from a large number of noisy patches in the 1/10-count frame image to their less noisy correspondences in the full-count frame image. The training patches consisted of a vast number of structural patterns with a range of noise levels, which made the ANN model serve the same purpose as a Gaussian kernel but perform as an anisotropic kernel. Therefore, the trained ANN model could be reasonably applied multiple times to further denoise an image. As anticipated, applying the trained ANN model multiple times reduces more noise while introducing smaller bias than the Gaussian kernel with matching noise reduction capability.

The cascade testing scheme also facilitates the evaluation of the denoising property of the ANN and ANN+HYPR methods in terms of noise versus bias tradeoff, as shown in figures 3, 4, 6, and 7. To investigate the effect of application times on the ANN and ANN+HYPR methods, we applied the trained model ten times to the dynamic frames of a testing subject. For comparison, ten smoothing parameters were also applied to the Gaussian filtering, NLM-ST, and HYPR methods. The quantitative evaluation results from both dynamic frames and parametric images are summarized in table S1 and figure S6. As well demonstrated in figures 4 and 7, more applications or larger smoothing parameters reduce the noise more while introducing more bias for all the methods and the ANN+HYPR method displays its clear advantage in noise versus bias tradeoff. Taking the denoising introduced bias into consideration, we chose to apply the trained ANN model three times in the cascade scheme because the noise reduction change also slows down when the application number increases.

Furthermore, we compared the proposed cascade testing ANN with the cascade training ANN. The cascade training is similar to the cascade convolutional neural network (CNN) training scheme, which was applied to denoise the x-ray low-dose CT images (Wu *et al* 2017). The cascade training strategy was implemented by feeding the output of the first trained CNN, i.e. the processed training dataset, as the input of the second CNN with the same structure to initiate a new training, and the same way was applied to the following cascades, which resulted in multiple different CNN models. The cascade strategy in both training and testing phases aimed to remove artifacts induced by the denoising when patterns rarely seen in the training dataset or too strong noise were encountered. We could also train (and apply) the ANN model multiple times similarly, which would be different from the proposed method in which the model was trained once and applied three times. In the cascade training ANN scheme (as shown in figure S7(a)), the ANN trained once has removed noise significantly in the processed training frame. Training the ANN model second and/or third times with the denoised frame, therefore, would not result in as much noise reduction capability as the first ANN model. Nevertheless, we have used the same subject to extract patches as the training data to train the three models in the cascade training scheme. For thorough comparison, the three models were also trained each with patches extracted from different subjects, respectively. The comparison of results from the proposed cascade application and the would-have-been cascade training ANN model are shown in the figure S7 (b1)-(c2) to justify the selection.

### 5.4. Generalization capability

As stated earlier, the large number of training image patches extracted from a dynamic frame of the training subject can capture local features containing structural patterns such as edges, smooth regions, textures, and a range of noise levels, which contributes to the generalization capability of the trained model. This is based on the fact that all possible images can be constructed using noise and structural patterns mentioned above (Elad and Aharon 2006). In addition, the normalization steps during training and testing enable the trained model to process the patches extracted from different testing subjects. In our study, the testing data include different image frames with different noise levels of various subjects with normal or Parkinson’s disease diagnosis, genders, weight, and injected dosages, acquired on a same scanner. Applying the trained ANN model to the aforementioned data, we demonstrate that the proposed patch-based ANN framework improves the noise versus bias tradeoff in data which are not included in training. In contrast to the ANN-based denoising methods using temporal information or CNN-based algorithms, which require multiple number of training subjects and result in erroneous outcomes when applied to data not included training, the proposed framework has the advantage of requiring one training subject and generalizing well in all testing subjects.

It may not reach similar promising results shown in this study if we directly test the trained ANN model on data acquired with different tracers at different activity levels or other scanners of different resolutions. However, the low requirement of training data for the ANN model and the straightforward implementation of the ANN+HYPR framework will make it easy to adapt the proposed method to other tracers or scanners by re-training the ANN model with the acquired data in a specific situation. We plan to evaluate the proposed method in other dynamic PET imaging applications such as ^82^Rb myocardial perfusion imaging in the near future. We are also interested in studying the effectiveness of the proposed method in clinical tasks, with ongoing applications including enhancing separability between PD and HC subjects and detecting longitudinal disease progression from reduced-dose SV2A PET imaging.

## 6. Conclusion

We developed an ANN+HYPR denoising framework to improve dynamic SV2A PET imaging with reduced dose of ^11^C-UCB-J. The ANN model trained by a dynamic frame of a HC applied well to enhance all dynamic frames of other HCs and PD patients. Making use of the entire dynamic imaging data, we integrate the straightforward HYPR algorithm on spatial ANN processed image series to accomplish the spatiotemporal noise reduction task. This technique substantially suppresses noise while introducing minimal bias in the dynamic frames reconstructed from reduced count data and the resulting parametric images. Achieving a noise reduction of 70% in the distribution volume images, the ANN+HYPR method demonstrated the potential of decreasing the injection dose to 1/11 of the current amount for dynamic SV2A PET imaging.

## Data Availability

The data cannot be made publicly available upon publication because they contain sensitive personal information. The data that support the findings of this study are available upon reasonable request from the authors.
